# Supplementary material for: Convergent validity of video-based observer rating of drowsiness, against subjective, behavioral, and physiological measures
Source: PLoS One. 2023 May 8;18(5):e0285557. doi: 10.1371/journal.pone.0285557 (PMC10166535; doi:10.1371/journal.pone.0285557)
Supplement: S1 Table — Table 1 was translated from this Japanese version. (PDF) [file pone.0285557.s001.pdf]

ORD level (points)	Drowsiness description (Japanese)	Drowsiness description	Typial observed behaviour (Japanese)	Typial observed behaviour
D1 (1)	全く眠くなさそう	Not drowsy	・視線の移動が速く、頻繁である<br>・動きが活発で身体の動きを伴う<br>・目の開閉の動きが速い<br>・瞬きの周期は安定している<br>・体の動きが機敏	Fast and frequent eye movements<br>Frequent body movements<br>Eye blinks with fast eyelid movements<br>Regular time intervals between eye blinks<br>Active body movements
D2 (2)	やや眠そう	Slightly drowsy	・視線移動の動きが遅い<br>・唇が開いている<br>・視線移動が少ない<br>・目が垂れてくる	Slow saccadic eye movements<br>Lips open<br>Infrequent eye movements<br>Drooping eyelids
D3 (3)	眠そう	Moderately drowsy	・瞬きはゆっくりと頻発<br>・口の動きがある<br>・座り直しあり<br>・顔に手をやる<br>・目をパチパチさせる<br>・目の開きが小さくなる<br>・あくびが見られる<br>・疲れたような表情	Frequent slow eye blinks<br>Mouth movement<br>Sitting position change<br>Touching face<br>Frequent eye blinks<br>Less than half obscured pupils<br>Yawning<br>Tired-complexion
D4 (4)	かなり眠そう	Very drowsy	・意識的と思われる瞬きがある<br>・あくびは頻発し、深呼吸も見られる<br>・頭を振る、肩の上下運動などの無用な身体全体の動きあり<br>・瞬きも視線の動きも遅い<br>・ぼーっと1点を見つめる<br>・焦点が定まらない<br>・黒目が半分以上隠れる	Voluntary eye blinks<br>Frequent yawning and deep breathing<br>Unnecessary body movements such as shaking of the head, up and down movement of the shoulders, etc.<br>Slow blinking and SEM<br>Staring blankly at a single point<br>Inability to focus
D5 (5)	非常に眠そう	Extremely drowsy	・瞼を閉じる<br>・頭が前に傾く<br>・頭が後ろに倒れる<br>・頬が落ちる（たるむ）	Closed eyes<br>Forward tilted head<br>Backward tilted head<br>Sagging cheeks
S (5)	居眠りしていそう	Sleeping	・D5状態が継続し覚醒することがないなど「居眠りしていそう」と判断される状態	D5 continues and does not wake. The state is judged as "likely to be asleep".

ORD, Observer Rating of Drowsiness; SEM, Slow Eye Movement
