# Supplementary material for: Convergent validity of video-based observer rating of drowsiness, against subjective, behavioral, and physiological measures
Source: PLoS One. 2023 May 8;18(5):e0285557. doi: 10.1371/journal.pone.0285557 (PMC10166535; doi:10.1371/journal.pone.0285557)
Supplement: S2 Table — This KSS was translated from the English version of the scale in EU Law, to approve driver drowsiness and attention warning (DDAW) [41]. (PDF) [file pone.0285557.s002.pdf]

KSS	Description of sleepiness in Japanese	Description of sleepiness
1	非常にはっきりと目覚めている	Extremely alert
2	とても目覚めている	Very alert
3	目覚めている	Alert
4	やや目覚めている	Rather alert
5	どちらでもない	Neither alert nor sleepy
6	眠くなる兆候がある	Some signs of sleepiness
7	眠いが、起きている努力は必要ない	Sleepy, no effort to keep awake
8	眠い、起きている努力が少し必要	Sleepy, some effort to keep awake
9	とても眠い、起きている努力がとても必要、眠気と戦っている	Very sleepy, great effort to keep awake, fighting sleep
