# Supplementary material for: Convergent validity of video-based observer rating of drowsiness, against subjective, behavioral, and physiological measures
Source: PLoS One. 2023 May 8;18(5):e0285557. doi: 10.1371/journal.pone.0285557 (PMC10166535; doi:10.1371/journal.pone.0285557)
Supplement: Attachment — Submitted filename: Response_to_Reviewers.docx [file pone.0285557.s005.docx]

**Response to Reviewers**

**We thank the editorial board and reviewers for their very helpful comments. Our manuscript has been carefully revised based on the review comments. The comments have greatly improved our manuscript. We have responded to each comment in a point-to-point manner, which is indicated in bold letters. The revised texts are highlighted in yellow in the Revised_Manuscript_with_Track_Changes.pdf file.**

**Responses to Reviewer #2:**

The manuscript is well written, but a couple of things need to be considered.

Comment 2-1. Why does the number of participants differ from what is mentioned in the abstract and in the methods section? In the abstract, you mentioned 17 participants, and in the methods, a total of 24 participants.

**Response:**

**Thank you for the constructive comments. The situation we encountered was that although the experiment initially involved a total of 24 participants, only 17 of them were analyzed. In the abstract, we concisely mentioned the analyzed number of participants as 17. However, we acknowledge that this may have caused confusion for the readers as it differs from the total number mentioned in the Materials and Methods section. Thus, to improve clarity and avoid contradictions, we have modified the first sentence of the Method section as follows:**

**“A total of 24 participants, aged between 20 and 29 years, were recruited by a subject recruitment service (OMRON EXPERTLINK Co. Ltd., Kyoto, Japan) from January to March 2021, and data from 17 of them were analyzed.”**

Comment 2-2. You included the inclusion criteria, but what were the exclusion criteria?

**Response:**

**As you pointed out in your comments, we have made revisions to the description of our participant recruitment criteria. We recognized that the exclusion criteria were not explicitly stated, leading to confusion. To address this issue and improve the overall clarity of the description, we have reorganized the criteria into separate sections for inclusion and exclusion, as shown below:**

**“All participants included in the study met the specified inclusion and exclusion criteria as follows. To avoid bias in both sleepiness and driving performance, the participants had to meet the following criteria: a score of less than 15 on the Center for Epidemiological Studies-Depression Scale, an intermediate type of morningness–eveningness questionnaire (MEQ), not having had any experience of shift work in the three months before the experiment, no travel to different time zones during the three months before the experiment, not using drugs more than double the administration interval, non-smoker, a body mass index (BMI) of lower than 25, and a score of less than or equal to 21 on the short motion sickness susceptibility questionnaire (MSSQ-Short) (to exclude the top 20% population who was susceptible to motion sickness). Additionally, participants had to possess a valid car driving license. The exclusion criterion was wearing glasses to measure eye movement. However, participants wearing contact lenses were eligible to participate.”**

Comment 2-3. In line 126, the procedure section, mention the city name.

**Response:**

**We appreciate your comment regarding the need to include the city name for readers who do not live in Japan. In order to address this issue, we have updated the sentence to include the city name as follows:**

**“The data collection for this study was conducted from January to March 2021 in a quiet meeting room at a hotel near Shin-Toyota station, located in Toyota City, Japan.”**

Comment 2-4. Consider rewriting the last sentence at line 375.

**Response:**

**To improve the clarity and logical flow of the paragraph containing line 375, we have rewritten it as follows:**

**“Further research is required to establish ORD as a useful ground truth. The main reasons ORD is not widely adopted are its time-consuming nature and labor-intensive nature. To address these challenges, ORD rating should be automated with artificial intelligence that can learn the association between ORD score rated by human raters and features extracted from facial videos. We believe that such an approach could greatly improve the usefulness of ORD as a ground truth for measuring sleepiness and enable its application in a broader range of settings.”**

Comment 2-5. References: The references are not appropriate. And consider changing the reference numbers 1, 2, 3, and 6. Please erase reference number 11.

**Response:**

**To address the issue you pointed out regarding the accessibility of references with numbers 1, 2, 3, and 6, which could not be accessed on the website of the International Transport Forum due to PDF files being replaced every fiscal year, we have removed those references and revised the corresponding sentences in the Introduction as follows.**

**“According to crash statistics, driver drowsiness accounted for 16% of all crashes in southwestern England in the United Kingdom that required police involvement, and 2.4% of vehicle crashes in the United States.”**

**This removal of these references does not change the meaning of the paragraph. In addition, as you mentioned, reference 11 has also been removed.**

**Responses to Reviewer #3:**

This is a well written paper. It is important to evaluate the Karolinska sleepiness scale in different countries or regions setting. I would suggest the authors to include some demographic description of the participants in supplementary materials.

**Response:**

**Thank you for the constructive comment. I have created a supplementary file named “S1_File.xls” containing the relevant data (sex, age, Morningness Eveningness Questionnaire, CES-D, BMI, MSSQ-short, sleep duration before the experiment, and wake-up time) for each participant. We have also added the following sentence at the end of the Participants subsection in the Results section:**

**“The demographic information for each participant is provided in the supplementary file (S1_File.xlsx).”**

**In addition, the file name of the supplementary data previously submitted was changed to S2_File.xlsx.**
